# Gestational hypoxia disrupts the neonatal leptin surge and programs hyperphagia and obesity in male offspring in the Sprague-Dawley rat

**DOI:** 10.1371/journal.pone.0185272

**Published:** 2017-09-28

**Authors:** Vladimir E. Vargas, Sunam Gurung, Benjamin Grant, Kimberly Hyatt, Krista Singleton, Sarah M. Myers, Debra Saunders, Charity Njoku, Rheal Towner, Dean A. Myers

**Affiliations:** 1 Departments of Obstetrics and Gynecology, University of Oklahoma Health Sciences Center, Oklahoma City, Oklahoma, United States of America; 2 Department of Cell Biology, University of Oklahoma Health Sciences Center, Oklahoma City, Oklahoma, United States of America; 3 Advanced Magnetic Resonance Center, Oklahoma Medical Research Foundation, Oklahoma City, Oklahoma, United States of America; Universidade do Estado do Rio de Janeiro, BRAZIL

## Abstract

The effect of gestational hypoxia on the neonatal leptin surge, development of hypothalamic arcuate nuclei (ARH) projections and appetite that could contribute to the programming of offspring obesity is lacking. We examined the effect of 12% O_2_ from gestational days 15–19 in the Sprague-Dawley rat on post-weaning appetite, fat deposition by MRI, adipose tissue cytokine expression, the neonatal leptin surge, ARH response to exogenous leptin, and αMSH projections to the paraventricular nucleus (PVN) in response to a high fat (HFD) or control diet (CD) in male offspring. Normoxia (NMX) and Hypoxia (HPX) offspring exhibited increased food intake when fed a HFD from 5–8 weeks post-birth; HPX offspring on the CD had increased food intake from weeks 5–7 vs. NMX offspring on a CD. HPX offspring on a HFD remained hyperphagic through 23 weeks. Body weight were the same between offspring from HPX vs. NMX dams from 4–12 weeks of age fed a CD or HFD. By 14–23 weeks of age, HPX offspring fed the CD or HFD as well as male NMX offspring fed the HFD were heavier vs. NMX offspring fed the CD. HPX offspring fed a CD exhibited increased abdominal adiposity (MRI) that was amplified by a HFD. HPX offspring fed a HFD exhibited the highest abdominal fat cytokine expression. HPX male offspring had higher plasma leptin from postnatal day (PN) 6 through 14 vs. NMX pups. HPX offspring exhibited increased basal c-Fos labeled cells in the ARH vs. NMX pups on PN16. Leptin increased c-Fos staining in the ARH in NMX but not HPX offspring at PN16. HPX offspring had fewer αMSH fibers in the PVN vs. NMX offspring on PN16. In conclusion, gestational hypoxia impacts the developing ARH resulting in hyperphagia contributing to adult obesity on a control diet and exacerbated by a HFD.

## Introduction

According to the Centers for Disease Control, obesity has reached epidemic levels with 34.9% of adults and approximately 17% of young adults in the United States considered obese (http://www.cdc.gov/obesity/data/adult.html). The genetic contribution to this alarming rise in obesity is relatively minor, implicating other causative factors. Barker [1] reported that a so-called ‘adverse intrauterine environment’ during the course of gestation was linked to the acquisition of cardiovascular disease and metabolic disorders (insulin resistance, type 2 diabetes, dyslipidemia and obesity) later in life. A number of maternal perturbations during gestation in experimental animals produce offspring exhibiting obesity and metabolic disorders or significantly increased the risk of these disorders by adulthood if the offspring are placed on high fat, high energy (“Western”) diets, supporting the epidemiological findings of Barker and colleagues. These maternal perturbations include maternal nutritional restriction, maternal obesity, and various maternal physiological or psychological stressors [2–8].

Hypoxia represents a major threat to the developing fetus often accompanying situations such as intra-uterine growth restriction (IUGR), preeclampsia, smoking, high altitude and obesity [9–16]. In rodents, moderate sustained gestational hypoxia (ranging from 10–12% O_2_) has been reported to program offspring for the development of both cardiovascular [17–19] and metabolic disorders [20, 21]. Recently, moderate sustained gestational hypoxia (11.5% O_2_ for the final six days of gestation) that resulted in IUGR pups in Sprague-Dawley (SD) rats was shown to increase abdominal adiposity and metabolic disorders (e.g. insulin resistance) in male offspring when fed a high fat diet (HFD) post-weaning [20, 21].

In adults, leptin plays a central role in governing food intake and energy expenditure [22]. Elevated plasma leptin, resulting from increasing adiposity, is associated with the development of hypothalamic leptin resistance, hyperphagia and contributes to obesity [23]. Developmentally however, leptin plays a neurotrophic role in terms of establishing the proper neuronal projections from the Arcuate Nuclei of the hypothalamus (ARH) to the other key hypothalamic nuclei (e.g. lateral hypothalamic area [LHA], ventromedial nuclei [VMN], paraventricular nucleus [PVN]) that comprise the appetite neurocircuitry [24]. Rodents exhibit a neonatal leptin “surge” during the first two weeks post-birth- a period during which the ARH neurocircuits are established in these species [25, 26]. Leptin appears essential for the development of the hypothalamic appetitive neurocircuitry during this neonatal period in rodents. However, both plasma concentrations of leptin achieved during this critical period, as well as the timing of the leptin surge impact development of appetite control [27–29]. Indeed, altered timing of the leptin surge, suboptimal or excess plasma leptin or leptin receptor antagonism during the surge period induces varying metabolic profiles by adulthood including hyperphagia [27–32].

The effect of gestational hypoxia on the neonatal leptin surge and programming of appetite remains unexplored. In a pregnant sheep model of sustained moderate gestational hypoxia, we found that late gestation fetal plasma leptin was elevated as well as leptin expression in fetal abdominal adipose [33] indicative that gestational hypoxia may impact leptin expression. In the present study, we explored the effect of moderate (12% O_2_), sustained (four days) hypoxia during late gestation (days 15 through 19) in the SD rat on: 1) the neonatal leptin surge, 2) αMSH projections to the paraventricular nucleus in neonates, 3) ARH response to exogenous leptin during the neonatal period, 4) post-weaning appetite, 5) fat deposition at adulthood, and 6) adipose tissue cytokine expression. At adulthood, fear/anxiety behavior and motor activity were assessed as these may affect food intake and fat deposition. We also compared the response of control and hypoxic male offspring to HFD vs control diets (CD).

## Materials and methods

### Animals procedures

All procedures were conducted with approval of the Institutional Animal Care and Use Committees at the University of Oklahoma Health Sciences Center and Oklahoma Medical Research Foundation, Oklahoma City, OK. Timed pregnant SD rats were obtained from Charles River Laboratories (Wilmington, MA) on day 10 of gestation. Upon arrival, the dams were pair-housed and allowed to acclimate to the animal facility environment with standard chow and water *ad libitum*.

### Gestational hypoxia general protocol

Following acclimatization, SD dams were placed (in pairs) in their cages into a hypoxic chamber (Cat. #. Manual Version 0.1 d0605, BioSpherix, Redfield, NY) and subjected to moderate hypoxia (Hpx 12% O_2_) starting at 0:800 h on day 15 through 0:800 h on day 19 of gestation. Normoxic controls dams were placed in hypoxia chambers with room air for the same time period at the same stage of gestation. Continuous hypoxia was achieved by delivery of nitrogen gas flow into the hypoxic chamber controlled by an oxygen sensor (Pro-Ox Model 110, Manual Version 1.0 d0207, BioSpherix, Redfield, NY) throughout the experimental procedure. From 19 days gestation to term, dams were single housed through delivery.

### Effect of gestational hypoxia on maternal food intake, fetal weight

Previous studies reported decreased food intake and fetal weights in rodents in response to 10% hypoxia during gestation [17, 19, 21, 34]. To assess the effect of our four day 12% O_2_ gestational hypoxia paradigm on maternal food intake, SD dams (n = 5) were subjected to moderate hypoxia (12% O_2_) starting at 0:800 h on day 15 through day 19. Timed pregnant SD dams (n = 5) treated as described above served as controls. At 24 hour intervals through day 19 of gestation, daily maternal food intake was determined. For this study, the hypoxic dams were returned to room atmosphere briefly each of the four days of hypoxia to weigh and replace food. Cages were checked for food pellets in bedding. The dams were then returned to hypoxic conditions.

To assess the effect of our four day 12% O_2_ gestational hypoxia paradigm on maternal food intake, SD dams (n = 15) were subjected to moderate hypoxia (12% O_2_) starting at 0:800 h on day 15 through day 19. Timed pregnant SD dams (n = 15) treated as described above served as controls. On days 19, 20 and 21, sets of dams (n = 5 dams per treatment group per day 19, 20 and 21 gestation) were euthanized with isoflurane anesthesia overdose, cervical dislocated and fetal weights recorded. Fetal weights were averaged for each dam and for statistics, n = 5 were used per group.

### Effect of moderate gestational hypoxia on offspring growth and appetite in response to a high fat or control fat diet

To assess neonatal growth, SD dams were subjected to moderate gestational hypoxia (n = 6 dams) or control normoxic (n = 6 dams) environments as described above. Litters were reduced to n = 8/dam for each group and male pups weights were obtained at 2, 6, 9, 16 and 21 PND.

To assess post-weaning weights, SD dams (n = 6/group) were subjected to moderate gestational hypoxia or control normoxic environments as described above. At birth, pups were culled from both hypoxic and normoxic dams to n = 8 pups/dam (when possible equally removing male and female pups) without cross-fostering. At three weeks of age, male offspring (2 males from each dam/diet) were randomly selected, weaned and provided either a control chow (20% kcal protein; 70% kcal carbohydrate, 10% kcal fat; Research Diets, Inc., New Brunswick, NJ) or a high fat chow diet (HFD; 20% kcal protein; 35% kcal carbohydrate, 45% kcal fat; Research Diets, Inc., New Brunswick, NJ) and water *ad libitum* through the remainder of the study (23 weeks). Offspring body weights were obtained once weekly throughout the study and 24-hour food intake was obtained once weekly during weeks 4 to 8, week 11 and week 23.

### Maternal pair-feeding paradigm

To discriminate between the effects of hypoxia and those potentially induced by the reduction in maternal energy intake, a group (n = 6) of timed pregnant SD dams under normoxic conditions were pair-fed the daily food allotment consumed by hypoxic dams from day 15 onward. For days 20 to 22 (term gestation) the pair fed dams were fed the amount of food consumed by the hypoxic dams on day 19. For this study controls included a group of normoxic, *ad libitum* fed dams (n = 6) as well as a group of hypoxic dams (n = 6). Upon birth, the litter size was reduced to 8 pups/dam (when possible equally removing male and female pups) and at weaning, 2 pups male from each dam (5–6 dams per group) were randomly selected were placed on normal rat chow through 13 weeks. Offspring body weights were obtained once weekly from weaning to week 13. Daily food intake was obtained once weekly during weeks 4 to 8 and at week 11.

### MRI of adult offspring

At 25 weeks post-birth, male offspring (n = 12 males per each group [2 males/dam]: normoxic control diet, normoxic HFD, hypoxic control diet and hypoxic HFD) were subjected to MRI at the Oklahoma Medical Research Foundation. Magnetic resonance images were acquired on a 7 Tesla (T); 30 cm horizontal bore USR Bruker system equipped with an AVANCE I console. A quadrature coil of 150 mm inner diameter and 266 mm in length matched and tuned to 300 MHz was used for pulse transmission and signal detection. A RARE (rapid acquisition with relaxation enhancement) image sequence was used with a repetition time of 1300 ms, an echo time of 15 ms, 25 contiguous horizontal slices of 3 mm thickness, a 180 mm × 100 mm field of view, and a 384×256 image matrix. Scans were acquired yielding a “water-suppressed” image that provided contrast between adipose tissue regions and regions having a typically low fat content, as previously described in Garteiser *et al*. [35]. For water suppression, a saturation pulse was used. A Mathematica (version 6.0; Wolfram Research, Champaign, IL, USA) notebook was developed to calculate the percent body fat/mouse body volume [35].

### Effect of moderate gestational hypoxia on offspring fear-anxiety behavior and motor activity as measured on an elevated plus maze

Male offspring (n = 2 per dam) from hypoxic or normoxic dams were fed a control chow diet post weaning to 16 weeks post-birth when they were subjected to elevated plus maze analysis of behavior as we have previously validated in our laboratory [36]. Briefly, the elevated plus maze is a measure of both fear/anxiety like behavior as well as a means to assess motor activity.

The elevated plus-maze was constructed of clear plexi-glass with two open arms and two enclosed arms (50X10 cm) at an elevation of 50 cm above the floor. Arms of the maze form a cross with two open arms being opposite each other. Light intensity on the maze was approximately 140 lux. Behavioral observations were recorded using a VCR in an adjacent room connected to a video camera mounted in the ceiling directly above the maze. The maze was cleaned with a 20% ethanol solution after use and allowed to dry completely between sessions.

Rats were placed in the center of the elevated plus-maze facing an open arm and their behavior analyzed for 5 minutes. Behavior on the elevated plus-maze was scored by an experimenter unaware of the treatment condition. Number of open arm entries (all four feet crossing the threshold of the open arm), percentage of open arm entries (open arm entries/total arm entries), time exploring the open arms, percentage of time exploring the open arms (time in the open arms/total time on arms) were determined as indices of anxiety by an experimenter unaware of treatment conditions. In addition, total number of arm entries, number of closed arm entries, and number of times the animal reared while on the maze were recorded as measures of locomotor activity.

### Quantitative real-time RT-PCR (qRT-PCR) mRNA for IL-1β, IL-6 and TNFα in peritoneal and subcutaneous adipose

At the termination of the study (25 weeks of age) adipose tissue was obtained. Sprague Dawley rats were placed in a glass chamber and euthanized rapidly with isoflurane overdose and tissues were collected from male offspring from hypoxic or normoxic control dams, rapidly frozen on dry ice and stored at -80°C until quantification of IL-1β, IL-6 and TNFα mRNA by qRT-PCR. We have previously described and validated the methods for qRT-PCR for a variety of genes in our laboratory [33, 37–39]. Total RNA was prepared from adipose tissue (n = 11 to 12 per group) with an RNA preparation kit as per manufacturer’s instructions (Qiagen, Inc.). Prior to reverse transcription, residual genomic DNA was removed from total RNA with DNase I (1 Unit, 60 min at 37°C; Ambion, Inc). The DNase I was subsequently removed from the RNA samples via PCR clean-up columns (Qiagen, Inc). An initial denaturation step was performed for 5 minutes at 95°C prior to first strand synthesis at 42°C for 50 minutes. Reverse transcription was then performed using one μg total RNA, with oligo dT as the primer, and Superscript II as reverse transcriptase; the reaction was terminated by heating to 70°C for 15 min.

Real time PCR was performed using 50 ng of cDNA (assumed equal to input RNA) per PCR. All PCR were performed in triplicate. Initial qRT-PCRs were performed using serial dilutions of cDNA ranging from 250 to 15.625 ng (250, 125, 62.5, 31.25, 15.625 ng) to determine that the quantity of cDNA used for analysis of each specific mRNA was within the linear range of amplification for each primer. For each mRNA, the starting amount of cDNA for qRT-PCR used was within the linear amplification range. For each primer set, the amplicon was directly sequenced by Sanger dideoxysequencing (Oklahoma Medical Research Foundation Sequencing Core, Oklahoma City, OK) to confirm amplicon identity. Sybr Green (1 X Sybr green master mix; Biorad, Hercules, CA) was utilized as the fluorophore and PCR was performed utilizing a Biorad iCycler equipped with the real-time optical fluorescent detection system. The primer sequences were derived from rat cDNA sequences obtained from the National Center for Biotechnology Information (NCBI). The NCBI accession numbers used are listed in Table 1.

**Table 1:** Forward (Fw) and reverse (Rv) primer sequences used for quantitative real-time primers.

GENE	Primer Sequence		NCBI Accession No.
**IL-1β**	**Fw**	5’ -AGGCTTCCTTGTGCAAGTGT- 3’	NM_031512
	**Rv**	5’ -TGAGTGACACTGCCTTCCTG- 3’	
**IL-6**	**Fw**	5’ -AGTTGCCTTCTTGGGACTGA- 3’	NM_012589
	**Rv**	5’ -ACAGTGCATCATCGCTGTTC- 3’	
**TNFα**	**Fw**	5’ -GAAACACACGAGACGCTGAA- 3’	NM_012675
	**Rv**	5’ -CAGTCTGGGAAGCTCTGAGG- 3’	
**CYCLO**	**Fw**	5’ -CCATCGTGTGTCAAGGACTTCAT- 3’	M19533.1
	**Rv**	5’ -CTTGCCATCTAGCCAGGGTCTT- 3’	

Fw, forward; Rv, reverse; cyclophilin (CYCLO); Interleukin 1β (IL-β;) Interleukin 6 (IL-6); Tumor Necrosis Factor α (TNFα); National Center for Biotechnology Information.

A three step PCR was used: 95°C for 45 sec, annealing (primer specific ranging between 55–60°C) for 30 sec and 72°C extension for 30 sec. A total of 35 cycles were performed. A melt curve analysis was conducted on each sample after the final cycle to ensure that a single product was obtained, and agarose gel electrophoresis confirmed that the single PCR product was of the expected size. We used cyclophilin as a ‘housekeeping’ mRNA, using the identical first-strand cDNA used for quantification of specific mRNAs of interest and in the same PCR run as for the gene of interest to circumvent any between run variation. As we previously reported [33, 40, 41] cyclophilin and GAPDH are equally efficacious when used as internal housekeeping mRNAs as they did not change under hypoxia or HFD conditions as demonstrated in our real time PCR application. There were no significant differences in cyclophilin mRNA levels between any treatment groups. Control PCR for each primer pair and RNA source included 1) elimination of reverse transcriptase during first-strand cDNA synthesis (assures that PCR product depends upon RNA) and 2) no RNA/cDNA in reverse-transcription reaction (assures that no amplicon contamination has occurred). Primers were utilized that provided 1) a single PCR product (identity confirmed by sequencing), 2) dilution curve of cDNA exhibited a slope of 100% ± 10% ‘efficiency’ where 100% = Δ3 Ct/log cDNA input (Ct is the threshold PCR cycle at which fluorescence is detected above baseline), and 3) the melt curve analysis post-PCR must demonstrate one product. For quantification purposes, a synthetic single stranded DNA standard was used to generate a standard curve (100, 10, 1, 0.1, 0.01 and 0.001 pg of standard DNA) for extrapolation of starting cDNA concentrations per reaction. Each standard point was run in duplicate and in the same PCR block as the unknowns. Linear regression was used to quantify starting RNA (cDNA) based on Ct values as extrapolated from the standard curve. The efficiency of the standard and primers was 100% based on the above criteria.

### Assessment of neonatal plasma leptin concentrations

To assess neonatal plasma leptin, SD dams were subjected to moderate gestational hypoxia (n = 5–6 dams per each day 6, 9 and 14 post birth) or control normoxic (n = 5–6 dams for each post-natal day) environments as described above. For assessment of leptin levels in neonatal male pups, plasma was collected from male pups (n = 3-to 5 per dam) on post-natal days (PND) 6, 9, and 14, (P0 is designated as the day of birth). For plasma collection, rat pups were placed in a glass chamber and rapidly anesthetized with isoflurane (USP, Butler Schein Animal Health, Dublin, OH). Pup weights were recorded and blood samples were obtained via cardiac puncture. Pups were then rapidly euthanized by decapitation. The plasma was rapidly frozen on dry ice then stored at -80°C until analysis. Plasma leptin was quantified using an ELISA assay kit for rat leptin, and following the manufacturer’s instructions (Millipore, Cat. # EZRL-83K, St. Charles, MO). The limit of sensitivity of this assay is 0.08 ng/mL (~5 pM) leptin. Plasma leptin concentrations were averaged for the pups from each dam for each post-natal day for statistical analysis.

### Recombinant leptin treatment and brain tissue collection

Sprague-Dawley dams were subjected to moderate gestational hypoxia or control normoxic environments as described above (n = 6 dams per group), and the pups culled to n = 8/dam at birth. On PND 16, recombinant rat leptin (R&D Systems, Cat. # 598-LP; 3μg/g body weight) or saline were administered via intraperitoneal injections. The concentration of leptin was based on reports in the literature (28, 29, 31) and after a preliminary study showing that maximal c-fos staining in control neonatal pups was obtained at 1 μg/g body weight. At 2h post-injection, pups were euthanized and the brains were removed and immediately fixed in 4% paraformaldehyde prepared in 0.1 M PBS (pH 7.4, Sigma, D8537, St. Louis, MO) for 48 hours at room temperature and stored at 4°C. Following fixation, the brains were immersed in 30% sucrose (J.T. Baker, Cat. # 4097–04, Center Valley, PA) prepared with PBS, 0.1 M, pH 7.4, and stored at 4°C for 48 hours. Following sucrose treatment, fixed brains were covered in embedding medium (OCT compound; Sakura Finetek Cat. # 4583, Torance, CA) and stored at -80°C. An n = 6 pups per group were analyzed; one male pup per dam/group.

### c-fos and αMSH immunostaining and image analysis

Brains were cryosectioned at 30 μm and sections were placed into cold cryoprotectant (0.1 M PBS, 30% sucrose, 30% ethylene glycol) and stored at -20°C until immunostained for c-fos to detect activated neurons and αMSH to assess ARH projections to the PVN. Every fifth section/animal containing hypothalamic ARH or PVN regions were selected. The ARH sections (at least three sections per pup) were immunostained for c-fos while the PVN containing sections (n = 3/pup) were immunostained for αMSH. Sections were removed from the cryoprotectant medium and washed 3 X 5 minutes each with 0.1 M PBS. Tissue sections were then treated with 2% hydrogen peroxide for 10 minutes, and washed with 0.1 M PBS. Following PBS washes the tissue was blocked for 1 hour on an orbital microtiter plate shaker in blocking buffer containing 0.1 M PBS, pH 7.4, 5% normal goat serum (Cat. # G9023, Sigma, St. Louis, MO), and 0.3% Triton X-100 (Cat. # X-100, Sigma, St. Louis, MO), at room temperature. After blocking the tissue sections were overnight incubated in either anti-c-fos (1:6000) primary rabbit antibody (Calbiochem, Cat. # PC38, San Diego, CA) widely recognized as a marker to detect activated neurons or αMSH (1:6000) to detect fibers in the PVN. Sections were then washed 3 times for 5 minutes each in 0.1 M PBS followed by goat anti-rabbit horse radish peroxidase secondary antibody (1:200) incubation (Invitrogen, Cat. # 656120, Frederick, MD) for 60 minutes on an orbital microtiter plate shaker at room temperature. Sections were mounted onto glass slides and allowed to dry overnight, then covered-slipped with paramount medium (Fisher Scientific, Cat. # SP15-100, Fair Lawn, NJ).

Digital images of the ARH were obtained at 20x objective magnification with an Olympus bright field microscope (Hitschfel Instruments B201 model BX40F, St. Louis, MO) equipped with a digital camera (Olympus DP11, St. Louis, MO). After subtraction of background, c-fos labeled neurons were identified in the images and counted using NIH Image J software (ImageJ1.46r, Wayne Rasband, NIH, USA; http://imagej.nih.gov/ij Java 1.6.0_29). For analysis of αMSH fibers in the PVN, the morphological limits of the PVN were visualized from the toluidine blue counter counterstained sections. A series (5 per each unilateral PVN; 10 total per each PVN per animal) of digital images (200 x 200 microns) were collected over the PVN and analyzed using NIH Image J64 following subtraction of background, the images were binarized and skeletonized using threshold parameters that optimized detection of labeled fibers. The total number of pixels in each binarized/skeletonized image was then determined and summed for each PVN and used as an index of the overall density of labeled fibers in each PVN. For all the histology analyses the images were performed blind to the treatment groups.

### Statistical analysis

Differences between moderate gestational hypoxia (12% O_2_) and control (room air) groups were determined using analysis of variance (ANOVA or 2-way ANOVA where multiple groups were compared [e.g. treatment x time]) with Bonferroni post-hoc tests or Student’s t-test where appropriate and p<0.05 was considered significant. All data are represented as means ± SEM.

## Results

### Effect of moderate gestational hypoxia on maternal food intake and fetal weights

**Fig 1:**
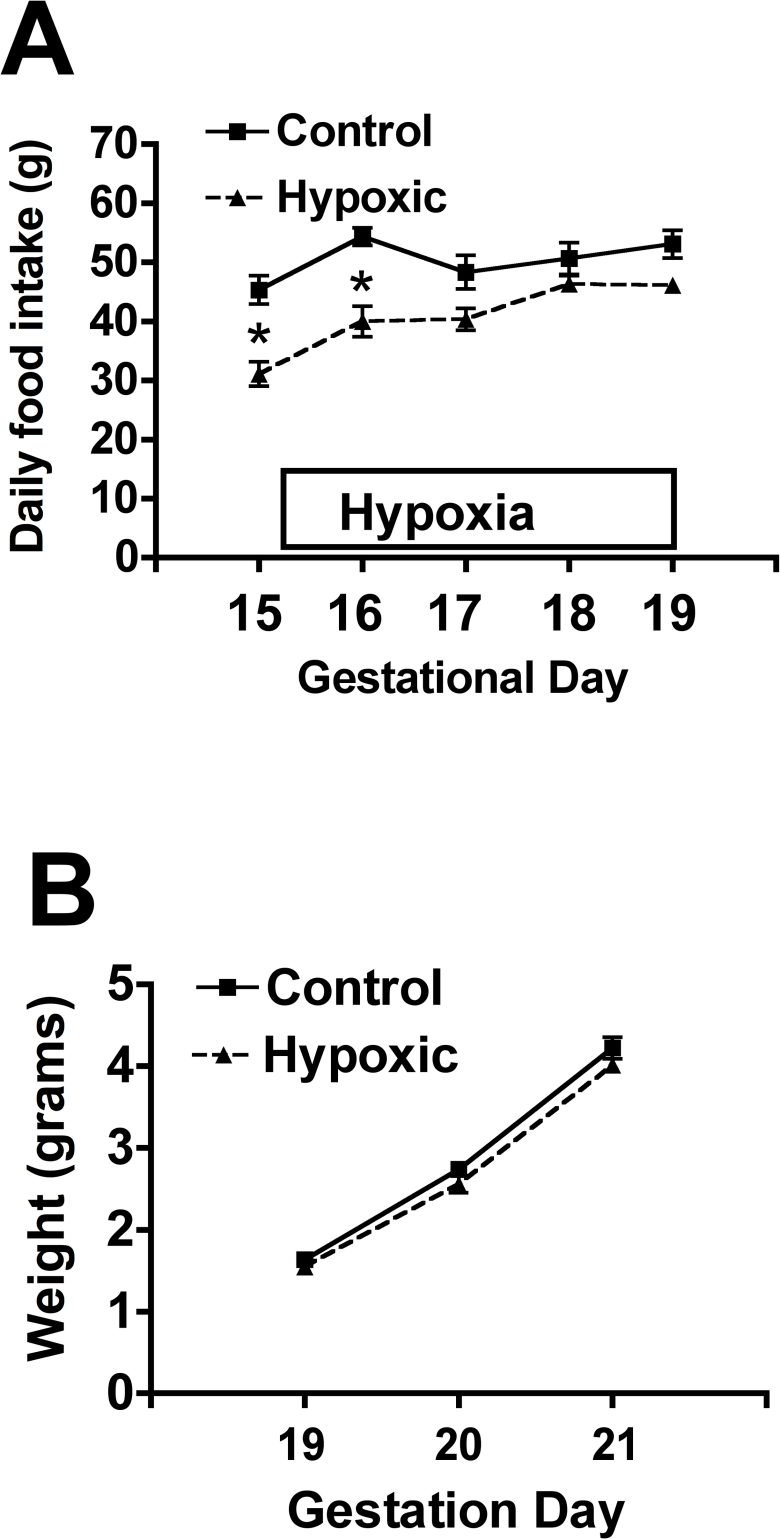
Effect of moderate gestational hypoxia on maternal food intake and fetal weight. **(A)** Exposure of pregnant SD rats to 12% oxygen (hypoxic; n = 5 dams) from 15–19 dG resulted in an initial decrease (p<0.05) in maternal food intake vs the normoxic group (n = 5 dams) during the initial two days of the hypoxic exposure (*, p<0.05). Maternal food intake in the hypoxic group was not significantly different compared to normoxic controls throughout the remainder of the hypoxic exposure. **(B)** Exposure of pregnant SD rats to 12% oxygen (hypoxic group) from 15–19 dG resulted in fetal body weights that were ~95% of normoxic fetuses (p<0.05; treatment effect) when measured at gestational days 19, 20 and 21 (Fig 2A; pup weights were averaged for each dam; n = 5 dams per treatment group).

Moderate gestational hypoxia (12% oxygen) from 15–19 dG resulted in an initial decrease (p<0.05) in maternal food intake in the hypoxic group on days 15 and 16 gestation when compared to the normoxic group (Fig 1A). Maternal food intake was not different compared to the normoxic controls throughout the remainder of the hypoxia treatment (days 17–19 gestation).

**Fig 2:**
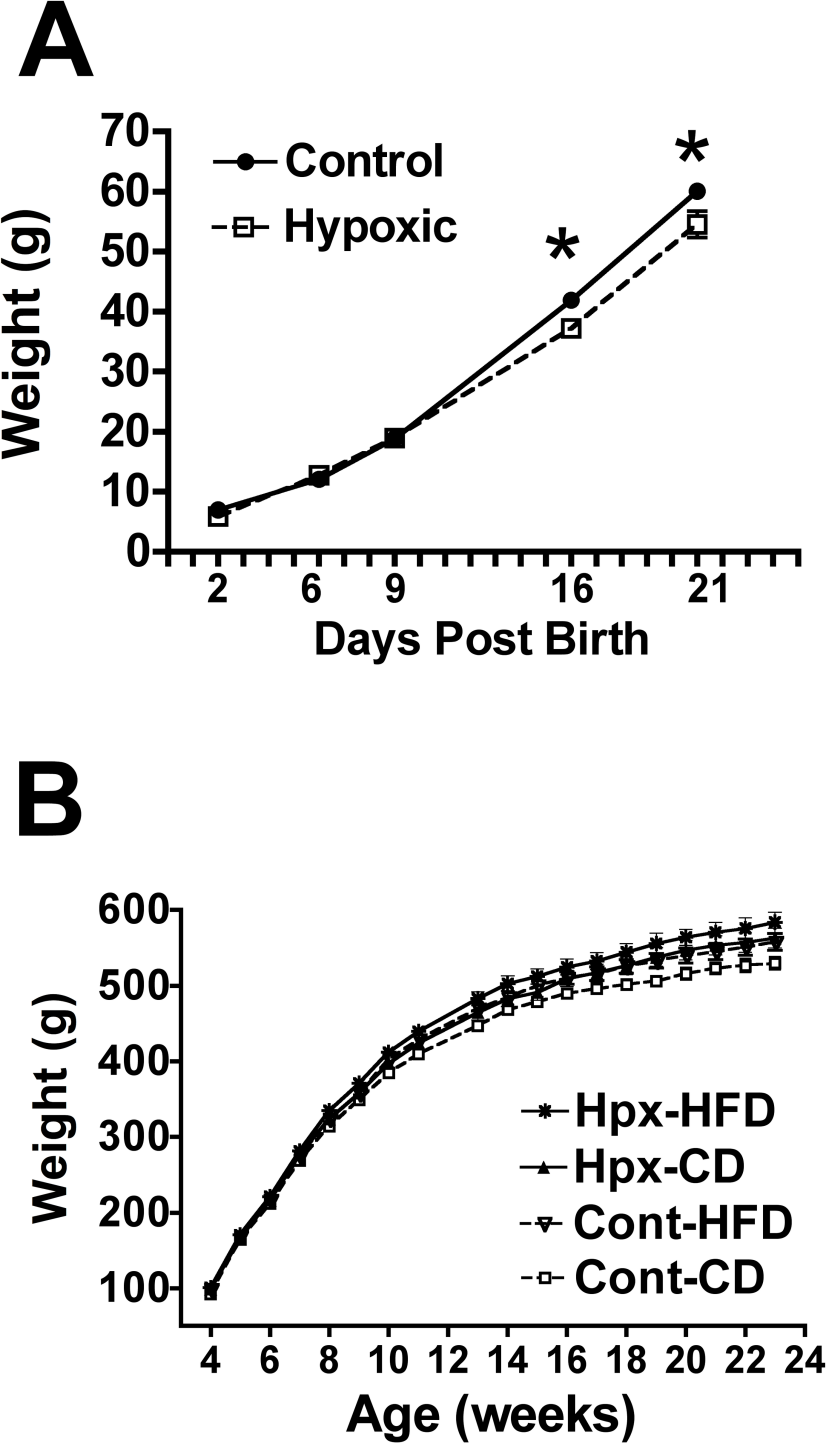
Effect of moderate gestational hypoxia on male offspring weight post-birth. **(A)** To assess neonatal growth, SD dams were subjected to moderate gestational hypoxia (n = 6 dams) or control normoxic (n = 6 dams) environments as described above. Litters were reduced to n = 8/dam for each group and male pups weights (n = 3 to 6 per dam) were obtained at 2, 6, 9, 16 and 21 PND. Male offspring born from dams exposed to moderate gestational hypoxia from 15–19 dG had similar body weights from 2 to 9 days post-birth; by 16 through 21 days post-birth however, the hypoxic offspring had significantly (*p<0.05) lower body weights vs the normoxic control group. **(B)** Male offspring born from dams exposed to moderate gestational hypoxia from 15–19 dG (Hypoxic; 12% oxygen) or normoxic control dams (Control) exhibited similar growth from 4 through 11 weeks of age, regardless of being fed a control chow diet (CD; 10% kcals from fat) or high fat (HFD: 45% kcals from fat) diet. From 13 weeks through 23 weeks of age, male offspring from hypoxic dams were significantly heavier compared to either control or hypoxic offspring fed the control diet or normoxic control offspring fed the HFD or the hypoxic offspring fed a control diet (n = 12 male offspring per group [n = 2 male offspring per dam, n = 6 dams per group]; p<0.05; ANOVA).

Moderate gestational hypoxia (12% oxygen) from days 15–19 of gestation resulted in fetal body weights that were ~95% of normoxic fetuses (p<0.05; treatment effect) when measured at gestational days 19, 20 and 21 (Fig 2A). However, posthoc analysis (Bonferroni) did not find significant differences between groups at any individual day of gestation. Similar to previous reports of gestational hypoxia, we noted no effects on litter size (number of pups/dam). Other studies applying more sustained (six-seven days) and/or severe hypoxia (10–11.5% oxygen; [17, 18, 21]) reported pup weights at birth being ~90% or less compared to normoxic controls and/or in the lower 15% of birth weights. Thus, although having significantly lower body weight, the fetuses in our study were below that of control fetuses, but not exhibiting IUGR. We did not observe a gender effect of hypoxia on fetal weight and thus combined males and females for final analysis.

### Effect of moderate gestational hypoxia on male offspring weight post-birth

From PND 2 through PND 10, there were no differences in pup weights (Fig 2A); however, when measured at PND 16 and PND 21, the hypoxic male offspring were significantly (P<0.05) lighter than normoxic controls (Fig 2B).

Body weights were not different between hypoxic or control male offspring fed either a high fat or control diet from week 4 through week 11 (Fig 2B). From weeks 13 through 23 weeks of age, hypoxic male offspring fed a control diet were significantly heavier compared to normoxic offspring fed the control diet. Hypoxic offspring fed a control diet and normoxic offspring fed a HFD were not significantly different at any age (Fig 2B). Hypoxic offspring fed a HFD were significantly heavier from week 13 onward compared to hypoxic offspring fed a control diet and control offspring fed either control or HFD.

### Effect of moderate gestational hypoxia on post-weaning food intake

**Fig 3:**
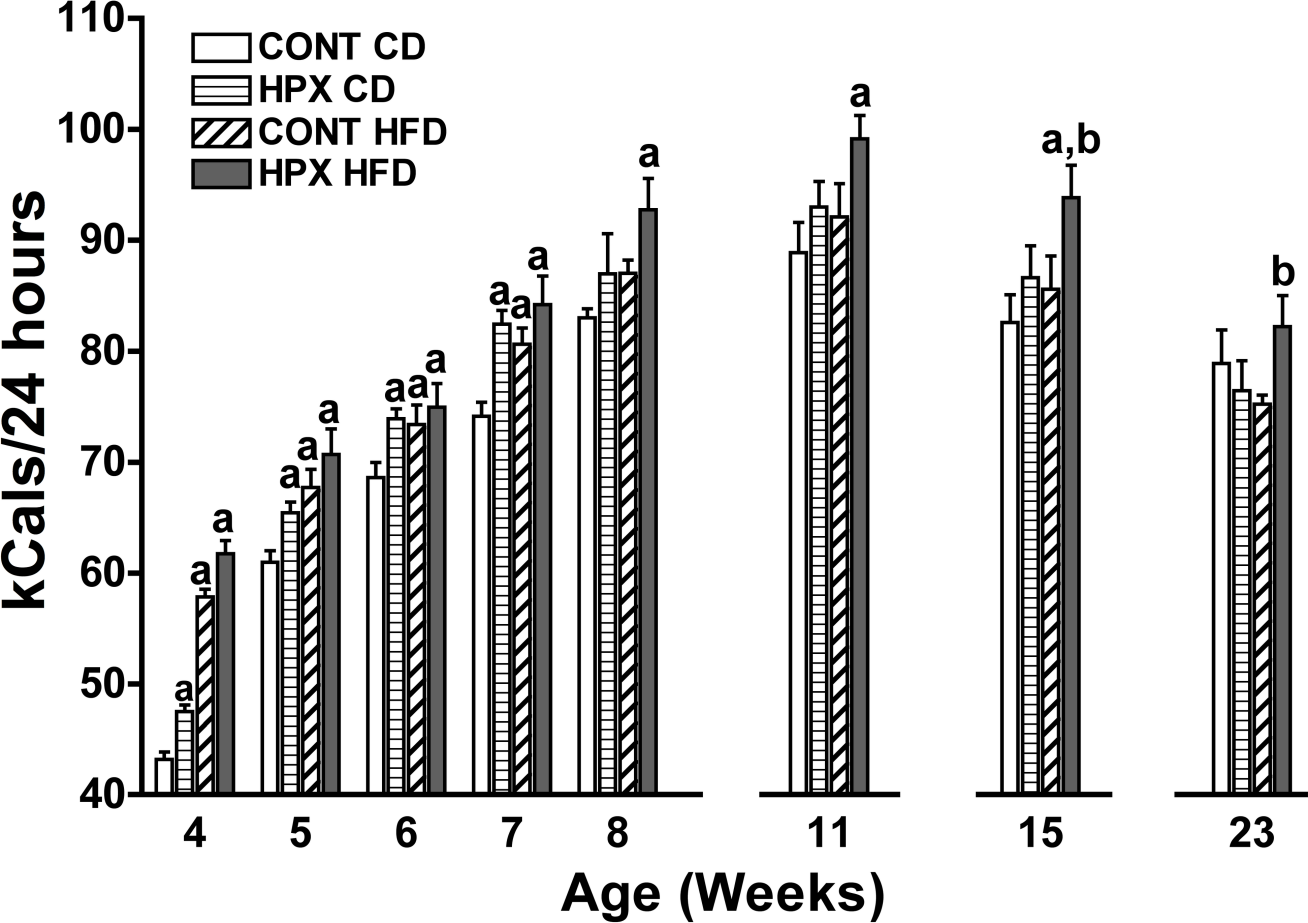
Effect of moderate gestational hypoxia on male offspring food intake post-weaning. Food intake (kCals/24 hours) was recorded from male offspring from weeks 4–8, and at weeks 11, 15 and 23. Juvenile food intake was significantly increased from weeks 4 to 7 ([a] p<0.05; ANOVA) in hypoxic male offspring fed either the HFD or the control chow diet (CD) compared to normoxic control offspring fed the CD. By week 8 post-birth, only the hypoxic male offspring on the HFD had significantly increased food intake ([a] p<0.05) compared to both normoxic control offspring fed either the CD or HFD. Similarly, at 11, 15 and 23 weeks of age, only the hypoxic male offspring on the HFD had significantly increased food intake (p<0.05) compared to both normoxic control offspring fed either the control chow or HFD ([a]; week 11 and 15) or compared to the normoxic offspring fed the HFD ([b]; week 23). (n = 12 male offspring per group; [2 male offspring per dam, n = 6 dams per group]).

From four to seven weeks post-birth a significant increase in food intake (kCal/24hrs) was observed in both hypoxic and normoxic offspring fed a HFD or hypoxic offspring fed the control diet compared to normoxic offspring fed a control diet suggesting hyperphagia in male juvenile rats born to dams exposed to the moderate gestational hypoxia when compared to control males (Fig 3). At 8 and 15 weeks post-birth, a significant increase in food intake was noted only in hypoxic offspring fed a HFD compared to normoxic offspring fed a control diet. By 23 weeks post-birth, hypoxic offspring on a high fat diet exhibited a greater food intake compared to control offspring fed a HFD; food intake in the hypoxic offspring on a HFD was not different from either hypoxic or control offspring on the control diet however.

### Effect of pair-feeding on offspring weights, growth and food intake

**Fig 4:**
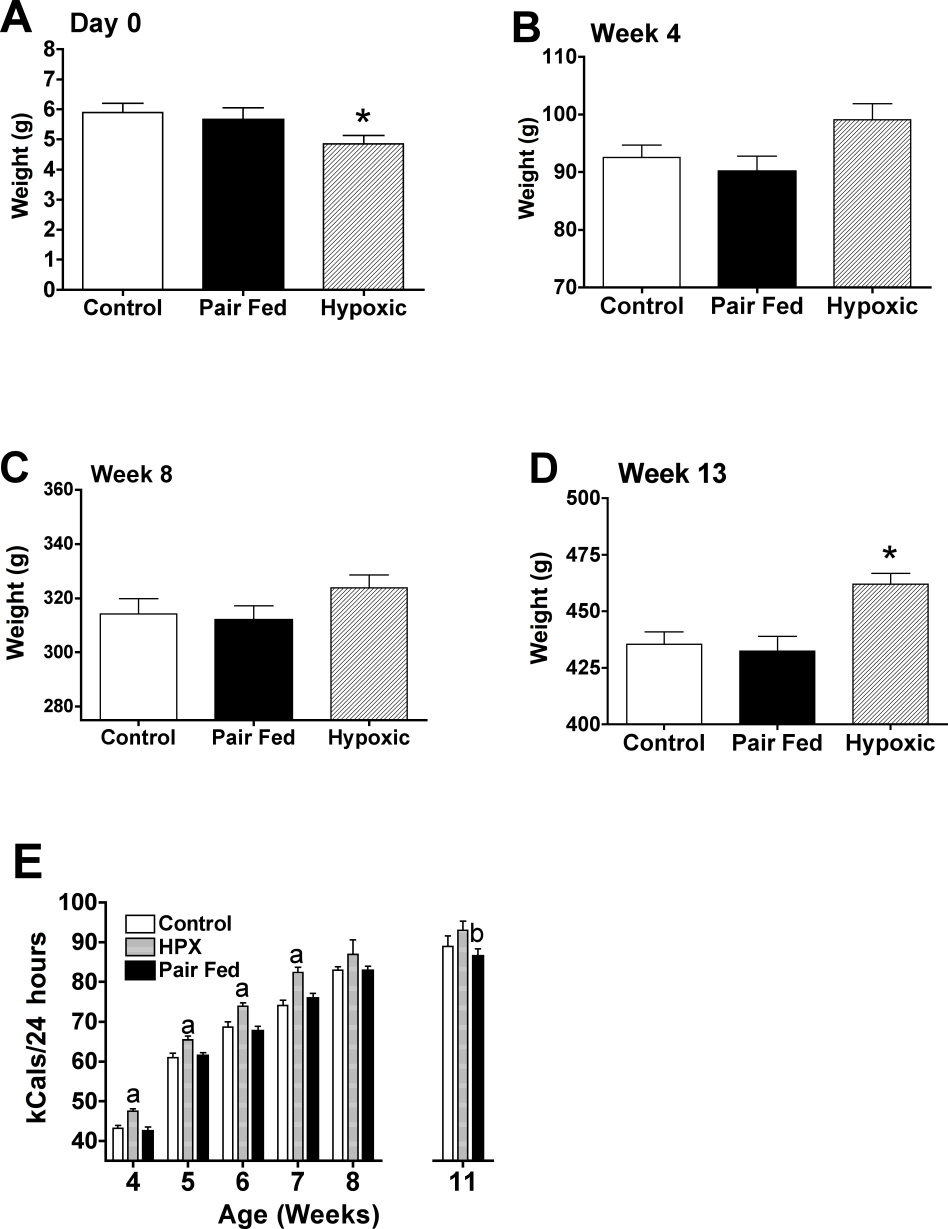
Effect of pair feeding on offspring weights at birth through early adulthood and post-weaning food intake. To discriminate between the effects of hypoxia and those potentially induced by the reduction in maternal energy intake, timed pregnant SD dams (n = 6) under normoxic conditions were pair-fed the daily food allotment consumed by hypoxic dams from day 15 onward. **(A)** Pup weights at birth were not different in pair fed dams (pup weights averaged per each dam; n = 6 pair fed, n = 5 hypoxic, n = 5 normoxic control dams); hypoxic pup weights were significantly (*p<0.05; ANOVA, Bonferroni’s posthoc test) less in pups from dams exposed to hypoxia compared to either control or pair-fed dams. **(B-D)** Male pup weights were not different between normoxic and pair-fed dams (n = 12 pair fed; n = 10 hypoxia; n = 10 control male offspring/group) a 4, 8 and 13 weeks post-birth. At 13 weeks, male offspring from the hypoxic dams were significantly greater (*p<0.05, ANOVA, Bonferroni’s posthoc analysis) compared to either control or pair-fed male offspring **(E)** Food intake in male offspring from normoxic control, pair-fed or hypoxic (HPX) dams fed a control chow diet post-weaning. Food intake was not different between male offspring from control and pair-fed dams throughout the study period while offspring from hypoxic dams had significantly ([a] p<0.05) greater food intake (kCals/24 hours) at weeks 4 through 7 compared to either control or pair-fed dams, while at 11 weeks, male offspring from pair fed dams had significantly lower food intake compared to those from hypoxic dams ([b] p<0.05).

Pups from pair-fed dams had birth weights (Day 0 post-natal) not different compared to normoxic control dams (Fig 4A). Similarly, male offspring from pair-fed dams had similar weights compared to normoxic control male offspring at 4, 8 and 13 weeks of age (Fig 4B, 4C and 4D). At 13 weeks of age, pair-fed male offspring were significantly lighter (p<0.05) compared to offspring from gestational hypoxia dams (Fig 4D). Food intake (kcals/24 hours) was similar between pair-fed male offspring and normoxic offspring through this period (Fig 4E) and less than male offspring from hypoxic dams from weeks 4 through 7 (p<0.05). At week 11, male offspring from pair-fed dams had less 24 hour energy intake compared to male offspring from hypoxic dams (p<0.05).

### Effect of moderate gestational hypoxia on fat deposition in offspring at adulthood fed a high fat or control diet

**Fig 5:**
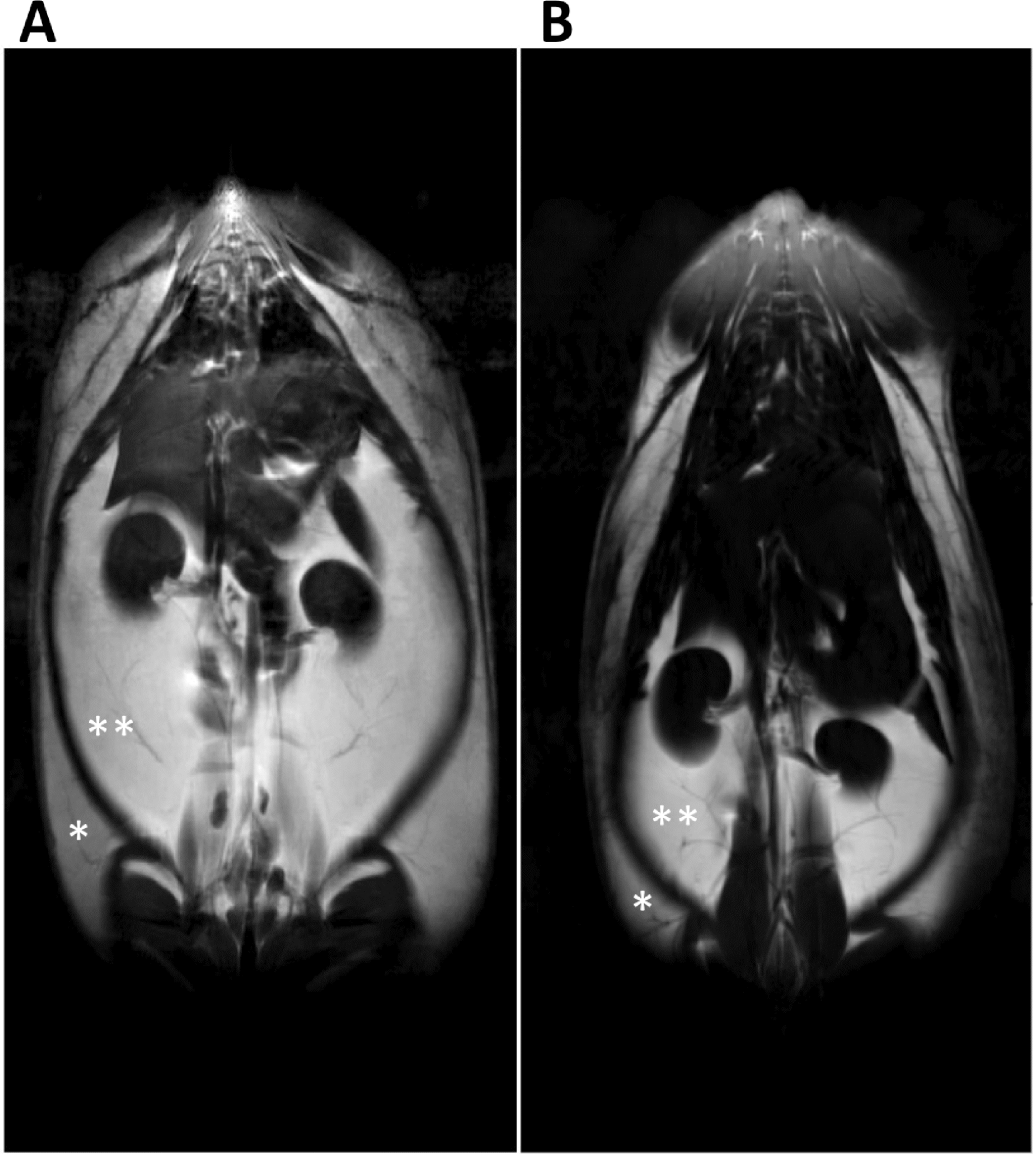
Water-suppressed MRI images at week 25 post-birth of fat distribution. In (**A**) male offspring from hypoxic dams fed the HFD and (**B**) male offspring from normoxic control dams fed the control chow diet. (**peritoneal fat; * subcutaneous fat).

**Fig 6:**
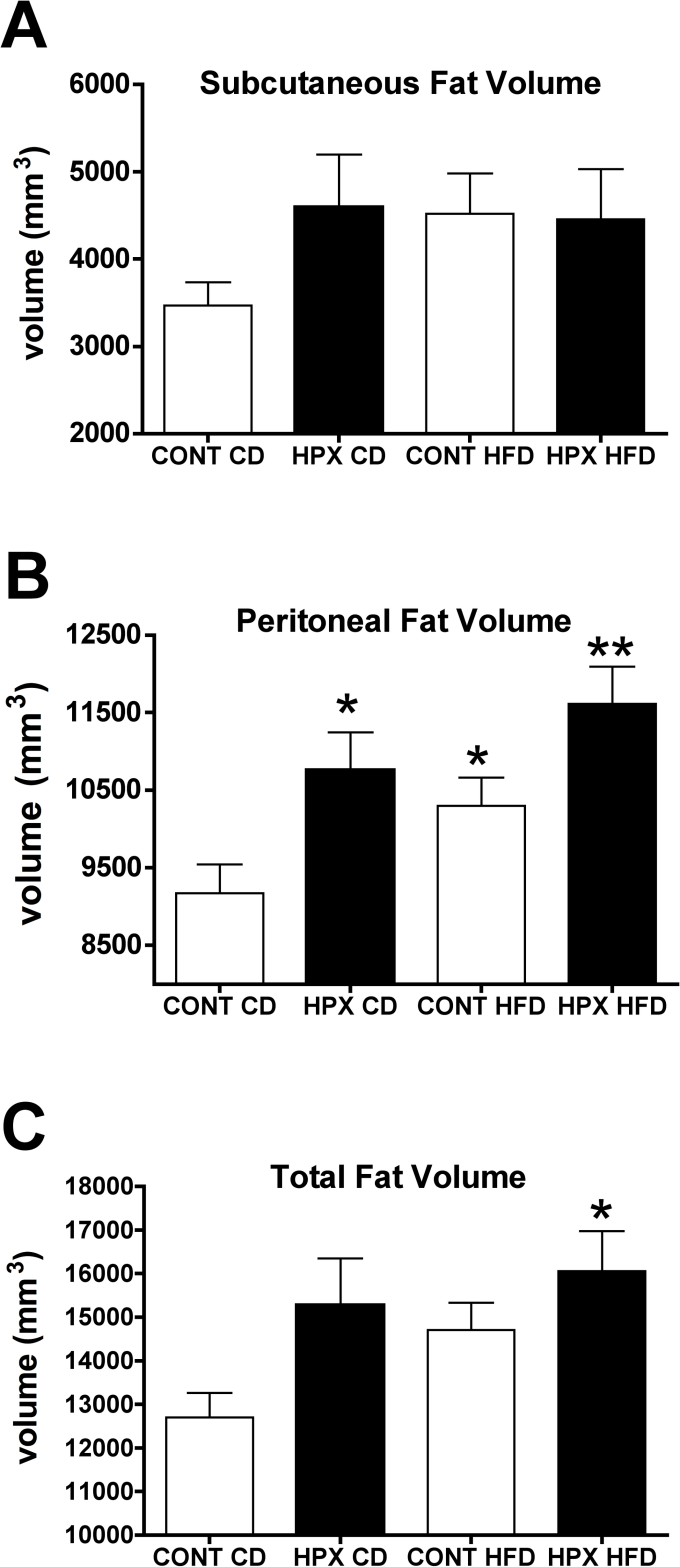
Effect of moderate gestational hypoxia on fat deposition in offspring at adulthood. Fat volumes (mm^3^) as determined by MRI for subcutaneous **(A),** peritoneal **(B)** and total (subcutaneous plus peritoneal) fat **(C)** in male offspring from hypoxic dams (HPX) and male offspring from normoxic control dams (CONT) fed either a control chow diet (CD; 10% kCals from fat) or a high fat chow diet (HFD; 45% kCals from fat) from 4 through 25 weeks post-birth (n = 11 to 12 per group). **(A)** There were no differences between groups in subcutaneous fat deposition. **(B)** Male offspring from hypoxic dams fed either the CD or HFD had significantly more peritoneal fat compared to male offspring from normoxic control dams fed the control diet (*, p<0.05). Male offspring from hypoxic dams fed the HFD exhibited greater peritoneal fat compared to male offspring from normoxic control dams fed the HFD (**, p<0.05). **(C)** Only the male offspring from hypoxic dams fed the HFD exhibited increased (*, p<0.05) total fat compared to the control male offspring fed the control diet. (ANOVA with Bonferroni’s post-hoc test).

There were no differences between groups in subcutaneous fat volume, although the hypoxic male offspring fed either the high fat or control diets and the normoxic offspring fed a HFD exhibited a trend (p = 0.06) for being greater (Figs 5 and 6A). Hypoxic male offspring fed either the control fat or the HFD had significantly increased peritoneal fat volume compared to normoxic male offspring fed a control diet (Fig 6B; p<0.05); the hypoxic offspring fed a high fat diet had significantly greater peritoneal fat compared to control normoxic offspring fed a HFD. Total fat volume (peritoneal plus subcutaneous) was only significantly greater in the hypoxic offspring fed the HFD (Fig 6C).

### Effect of moderate gestational hypoxia on offspring fear-anxiety behavior and motor activity as measured on an elevated plus maze

Moderate gestational hypoxia from days 15–19 of gestation resulted in no significant differences on indices of anxiety-like behavior on the elevated plus maze as well as indices of altered motor behavior (total entries, closed arm entries or rearing on maze) in the male offspring (Table 2) at adulthood (16 weeks of age).

**Table 2:** Effect of gestational hypoxia on offspring anxiety-like behavior and locomotor activity.

*Anxiety-like behavior*	Normoxic	Hypoxic	*p*
Open arm entries	3.42 ± 0.47	2.72 ± 0.35	0.236
% open arm entries	28.5 ± 2.5	23.5 ± 2.9	0.223
Time in open arm (s)	72 ± 9	59.3 ± 14	0.483
% time in open arms	24 ± 3.2	19.8 ± 4.8	0.484
***Locomotor activity***			
Total arm entries	11.64 ± 0.77	12.46 ± 0.83	0.48
Closed arm entries	7.67 ± 0.67	9.69 ± 0.86	0.07
Rearing	13.5 ± 1.9	14.7 ± 0.9	0.40

### Effect of moderate gestational hypoxia on IL-1β, IL-6 and TNFα mRNA in peritoneal and subcutaneous fat deposits in offspring at adulthood fed a high fat or control diet

**Fig 7:**
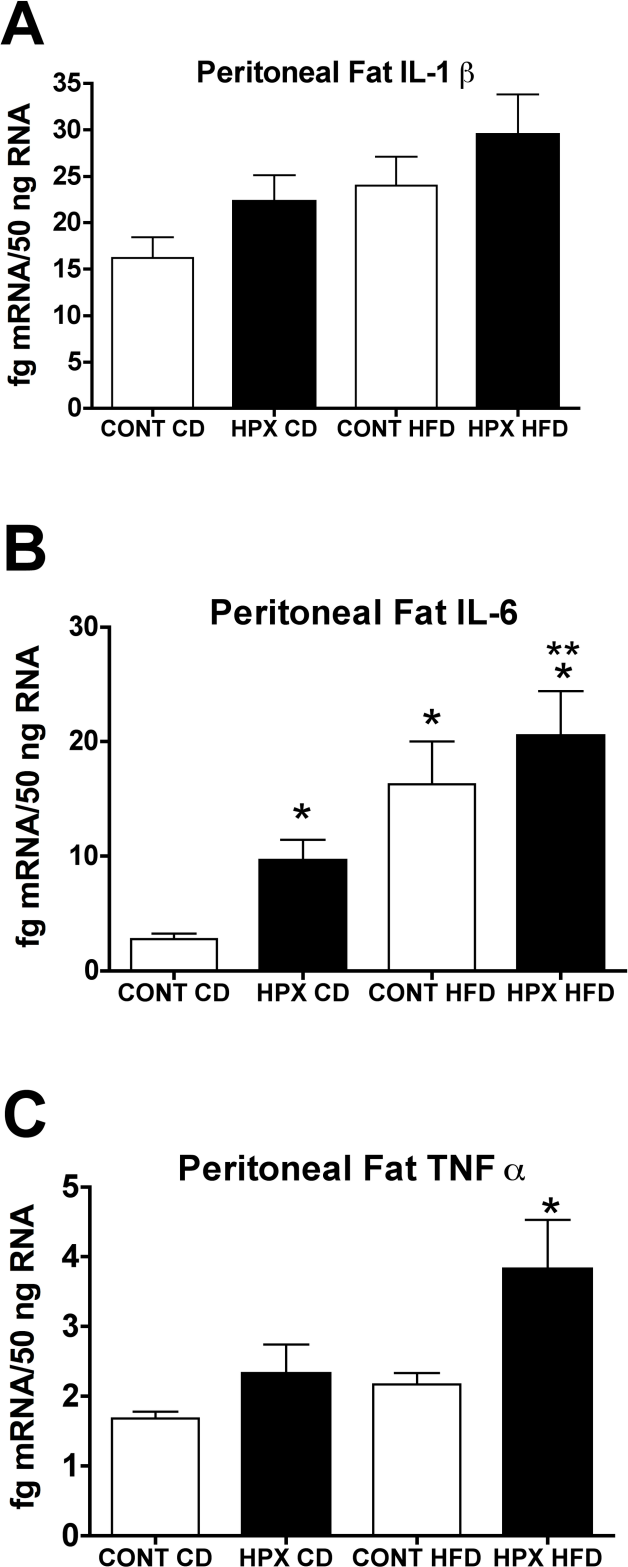
Effect of moderate gestational hypoxia on cytokines mRNA in peritoneal fat at adulthood. Messenger RNA for IL-1β, IL-6 and TNFα in peritoneal fat from male offspring born from hypoxic dams (HPX) and male offspring born from normoxic control dams (CONT) at 25 weeks of age. **(A)** IL-1β mRNA was significantly elevated in the peritoneal fat from male offspring born from hypoxic dams fed the HFD compared to male offspring born from normoxic control dams fed the control diet (CD; *, p<0.05). IL-1β mRNA in peritoneal fat from hypoxic male offspring fed a CD or normoxic control offspring fed a HFD was not different compared to normoxic male offspring fed the CD. **(B)** IL-6 mRNA was significantly elevated in the peritoneal fat from male offspring born from hypoxic dams fed either the CD or the HFD and in normoxic control male offspring fed the HFD compared to male offspring born from normoxic control dams fed the CD (*, p<0.05). IL-6 mRNA in the peritoneal fat from male offspring fed a HFD was elevated compared to hypoxic offspring fed the CD (**p<0.05). **(C)** TNF-α mRNA was significantly elevated in the peritoneal fat from male offspring born from hypoxic dams fed the HFD compared to male offspring born from normoxic control dams fed the CD (*, p<0.05). TNFα mRNA in peritoneal fat from hypoxic male offspring fed a CD or normoxic control offspring fed a HFD was not different compared to normoxic male offspring fed the CD. (n = 11 or 12/group; ANOVA with Bonferroni’s post-hoc test).

There were no differences in cytokine mRNA in subcutaneous fat between adult male offspring from hypoxic dams compared on the CD or HFD compared to control offspring from normoxic dams fed either of the diets. In peritoneal fat, only the adult male offspring from hypoxic dams fed a HFD had significantly elevated expression of IL-1β and TNFα (Fig 7A and 7C). IL-6 mRNA was significantly elevated in the peritoneal fat of both the adult control offspring fed a HFD as well as adult male offspring from hypoxic dams fed either the CD or the HFD (Fig 7B; p<0.05).

### Neonatal plasma leptin

**Fig 8:**
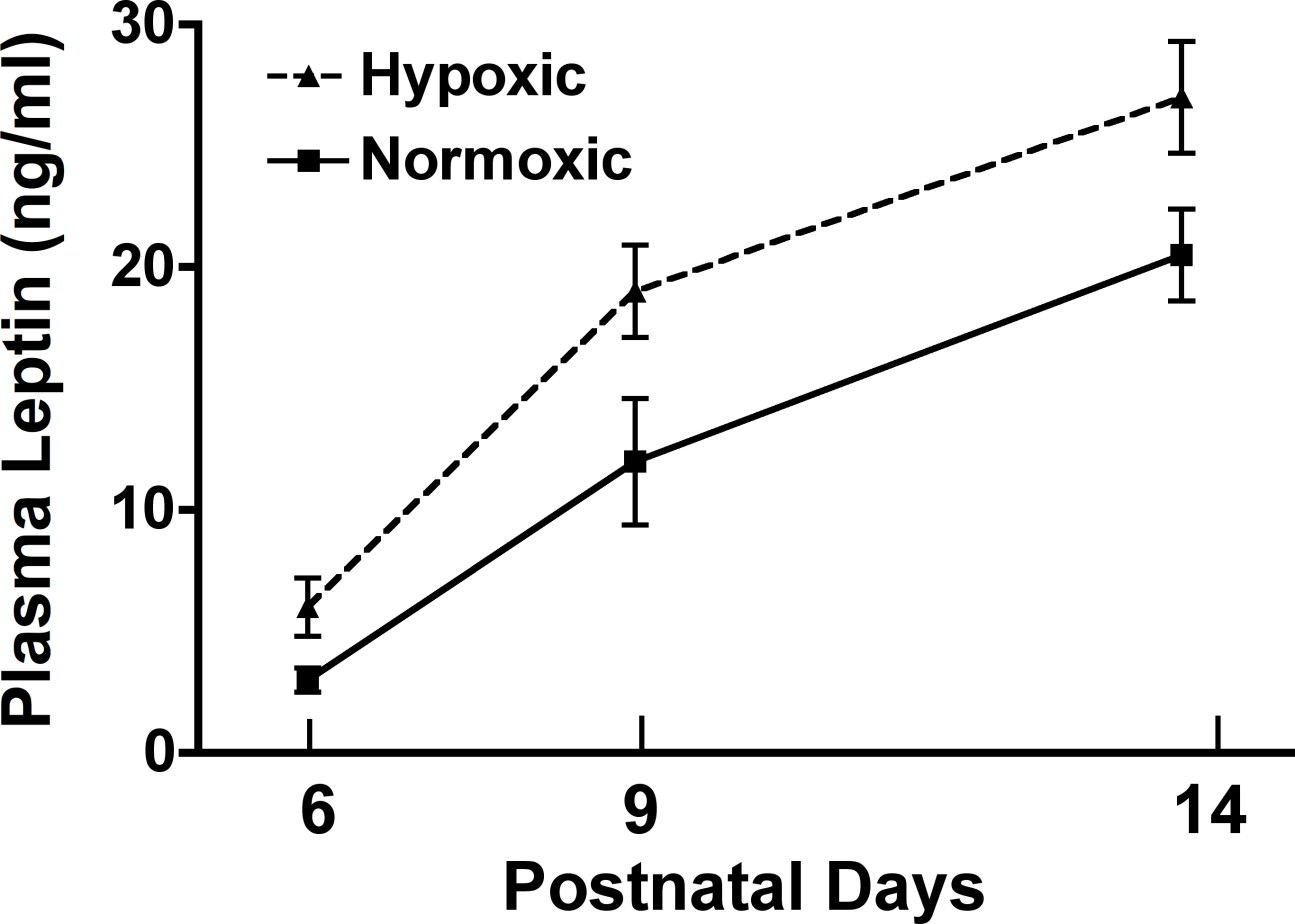
Neonatal plasma leptin from 6 through 14 days post-birth. Plasma leptin concentrations in male offspring from hypoxic dams and male offspring from normoxic control dams from 6 through 14 days post-birth. Plasma leptin levels progressively increased post-birth in both groups (time effect p<0.001). Interestingly, hypoxic male offspring had significantly greater plasma leptin (p<0.05 treatment effect; p<0.001, time effect), (ANOVA with Bonferroni’s post-hoc test).

Plasma leptin increased progressively from days 6 post-birth through day 14 post-birth in both pups born from normoxic control dams and hypoxic dams (Fig 8). Plasma leptin concentrations were significantly higher in the hypoxic pups compared to control pups over this period (p<0.05 treatment effect; p<0.001, time effect).

### Basal and leptin stimulated c-fos immunostaining in the arcuate nucleus of the hypothalamus at postnatal day 16

**Fig 9:**
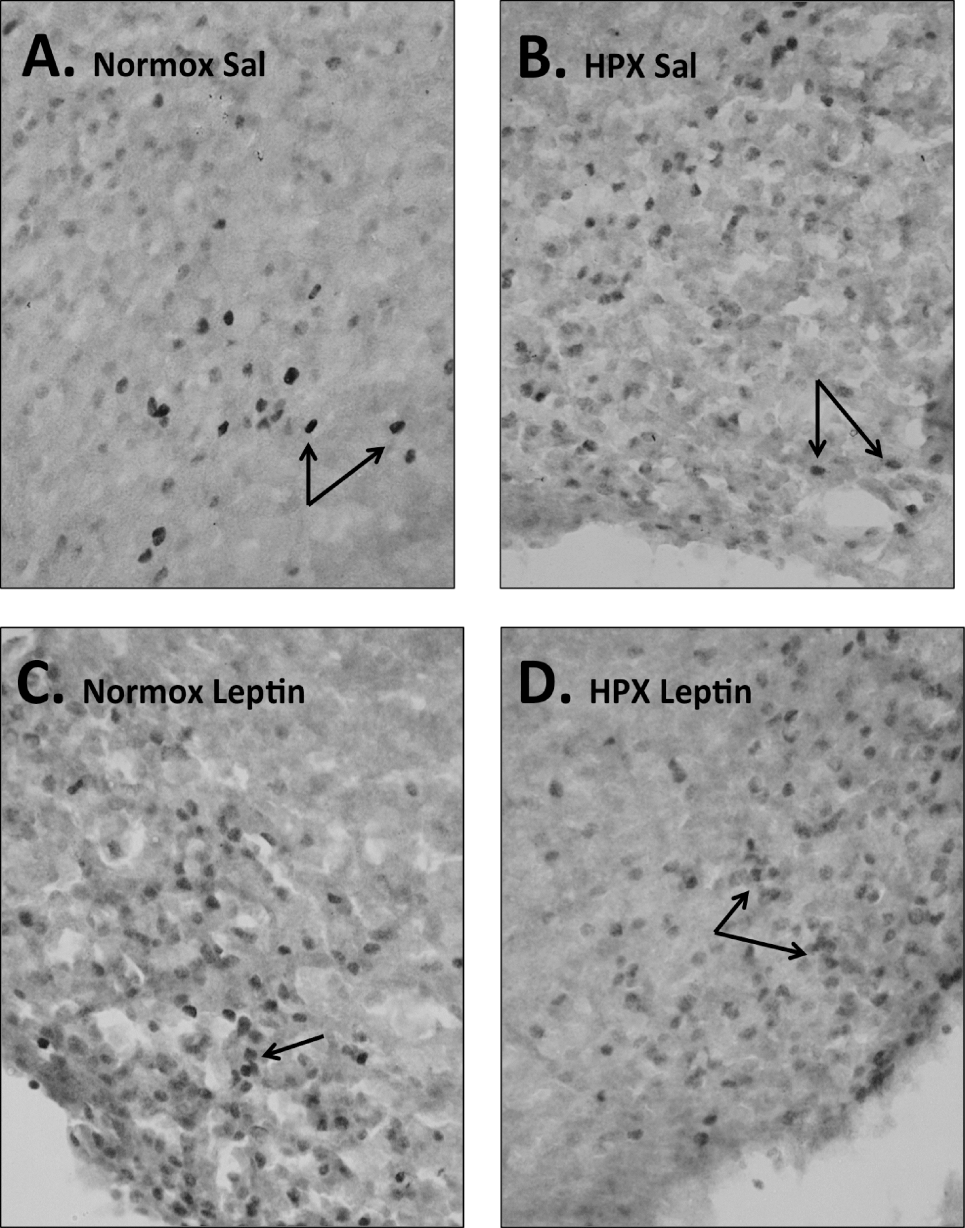
Hypothalamic ARH c-Fos immunostaining at postnatal day 16. Representative photomicrographs for immunostaining for c-Fos labeled neurons in the hypothalamic arcuate nuclei in normoxic control (Normox) and hypoxic (HPX) PND16 rat pups following administration of either saline (Sal; A, B) or leptin (C, D). Arrows denote labeled cells; n = 6 pups per group were analyzed; one male pup per dam/group.

**Fig 10:**
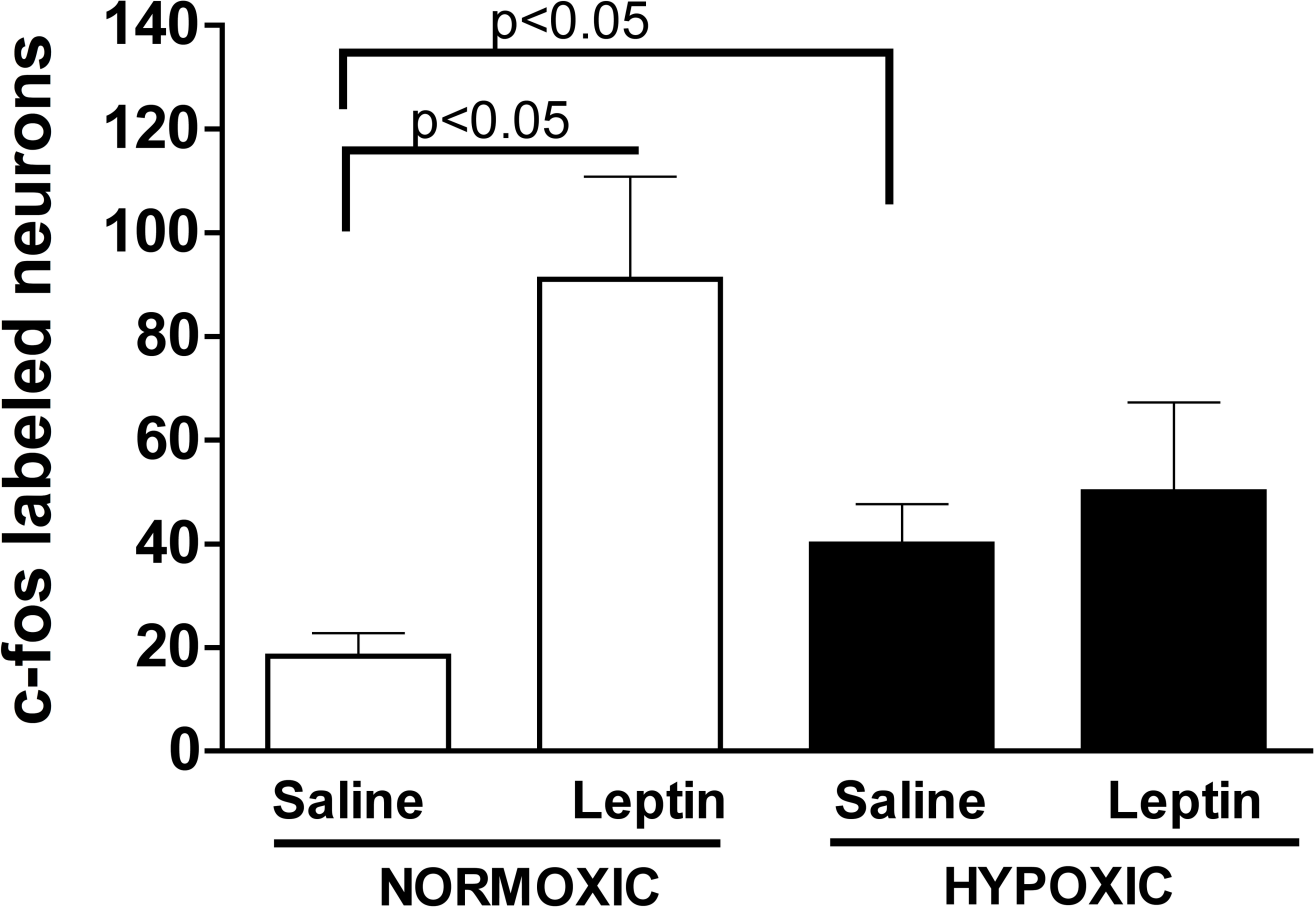
Basal and leptin-stimulated c-fos immunostaining in the ARH of the hypothalamus at postnatal day 16. Quantification of c-Fos in the hypothalamic arcuate nuclei (ARH) in male offspring from hypoxic or normoxic control dams at day 16 post-birth injected with either saline (Control) or recombinant rat leptin. There was a significantly greater number of c-Fos labeled neurons in ARH of male pups from hypoxic dams when compared to the normoxic control male pups under basal unstimulated (Saline) conditions (p<0.05). In response to leptin administration, a significant increase in the number of c-Fos labeled cells in the ARH was only observed in the male offspring from normoxic control dams (p<0.05). (ANOVA with Bonferroni’s post-hoc analysis).

There were significantly more c-fos labeled neurons in the ARH of PND 16 saline treated pups born from hypoxic dams compared to pups born from normoxic control dams (Fig 9A and 9B and 10; p<0.05). Leptin administration significantly increased the number of c-fos labeled ARH neurons in control pups (compared to control saline treated) but not in pups born from hypoxic dams compared to saline treated pups from hypoxic dams or control pups (Figs 9C, 9D and 10; p<0.05).

### αMSH projections to the hypothalamic paraventricular nucleus at postnatal day 16

**Fig 11:**
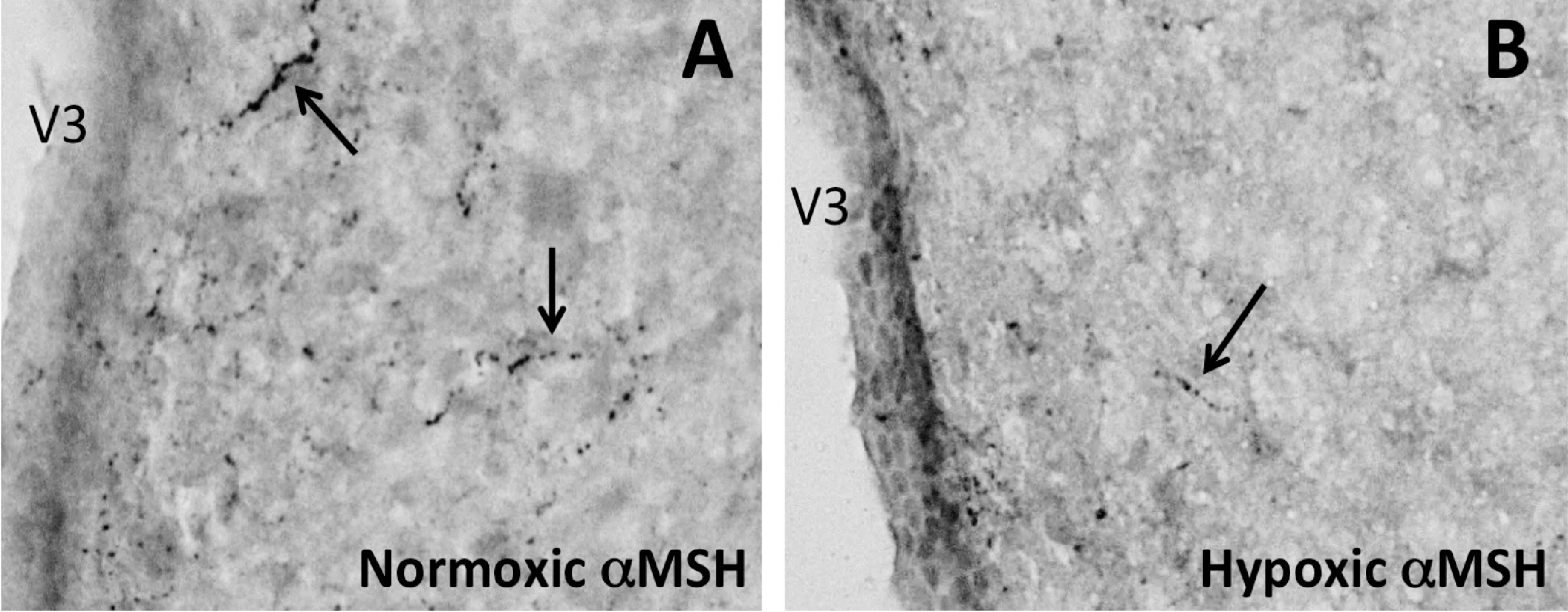
αMSH projections to the hypothalamic paraventricular nucleus at postnatal day 16. αMSH labeled fibers in the paraventricular nucleus of PN16 male offspring from normoxic control **(A)** and hypoxic **(B)** dams (n = 6 pups per group; one per dam).

**Fig 12:**
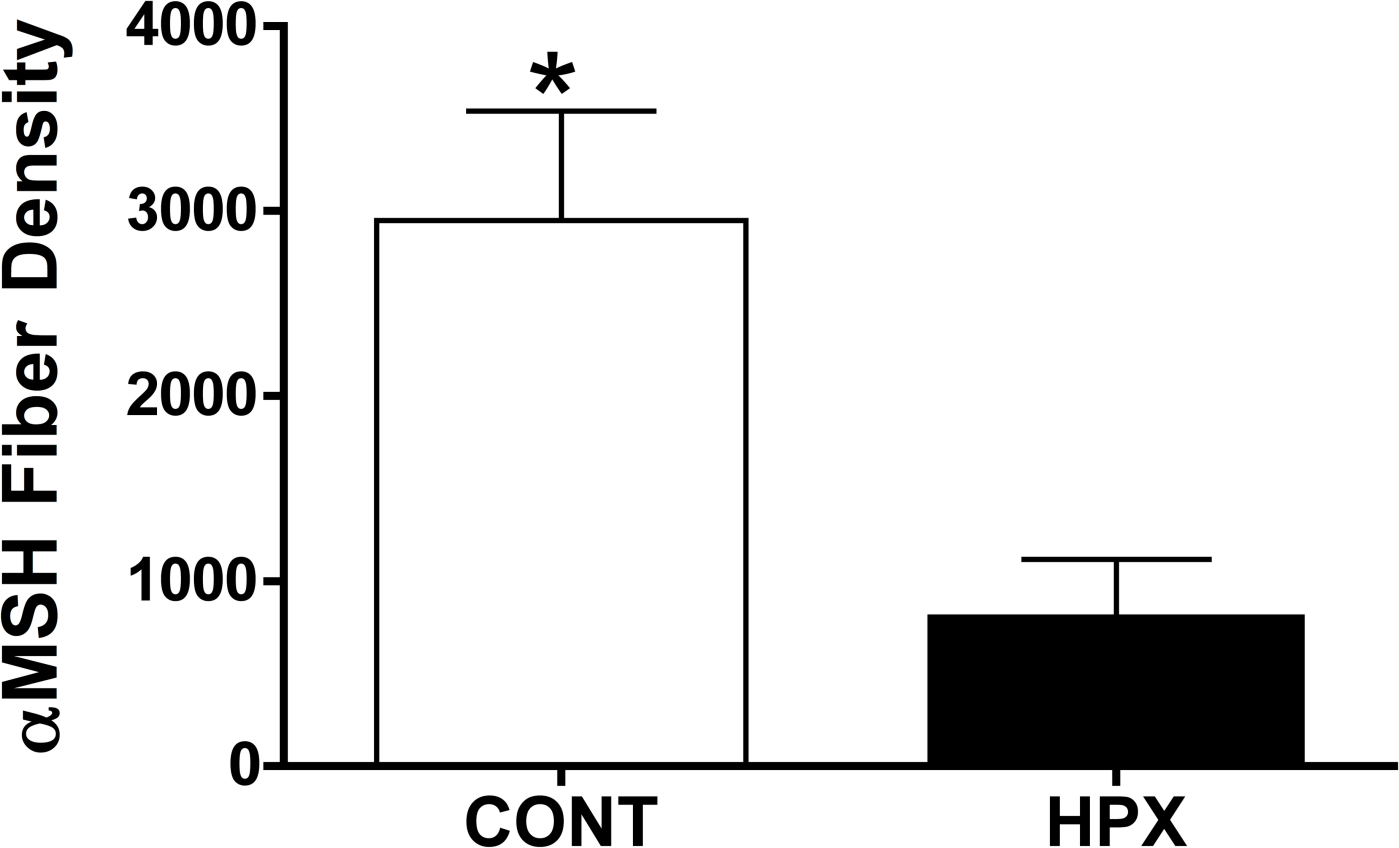
αMSH fiber density to the hypothalamic paraventricular nucleus at postnatal day 16. αMSH fiber density in the in the paraventricular nucleus of PN16 male offspring from normoxic control and hypoxic dams (n = 6 pups per group; one per dam). There was a significant decrease in αMSH fiber density (p<0.05) in male offspring born from hypoxic dams at PN16 compared to normoxic male offspring.

There were significantly fewer αMSH fibers in the PVN of pups born from hypoxic dams compared to control pups from normoxic dams (Figs 11A, 11B and 12; p<0.05).

## Discussion

Moderate gestational hypoxia is a common fetal perturbation, associated with a variety of pregnancy complications including preeclampsia, smoking, high altitude, obesity and placental insufficiency [9–16]. In the present study, we utilized a moderate hypoxia (12%) paradigm and limited the hypoxic exposure to a four-day window of late gestation (days 15 through 19 of gestation). While the fetuses were lighter on days 19–21 of gestation), they were still at ~95% of control fetal weight (Fig 2A). Further, post-birth their weights were not different from control pups until late in the suckling period when a small, albeit significant reduction in growth was noted. However, by a week post-weaning, the hypoxic male offspring weights had returned to that of controls. Thus, our model of moderate gestational hypoxia allowed us to more directly examine the effect of moderate gestational hypoxia in the absence of IUGR, and to focus on effects on appetite, post-weaning growth on either a control (15% kcals from fat) or high fat (45% kcals from fat) diet and to identify potential early mechanisms that could lead to perturbed appetite contributing to adult obesity.

### Effects of moderate gestational hypoxia on post-weaning growth and adipose deposition

There have been few studies focusing on the effects of gestational hypoxia in the programming of offspring appetite and adipose deposition, despite the common occurrence of this fetal perturbation in human pregnancies. The study of Rueda-Clausen et al [21], while finding similar body weights of male offspring exposed to gestational hypoxia compared to normoxic male control offspring fed either a control diet or when fed a HFD post-weaning to 11 weeks of age, noted increased abdominal adiposity in IUGR male offspring resulting from gestational hypoxia by early adulthood when fed high fat diet post-weaning. Similarly, Camm et al. [34] reported that gestational hypoxia (10% O_2_ from day 15–20 gestation) did not affect body weight at 16 weeks of age in male offspring fed a normal chow diet. Both of these studies did note signs of insulin resistance in the livers of the male offspring by early adulthood. In the present study, we found that the male offspring from hypoxic dams exhibited significantly greater weight gain post-weaning when fed a control chow diet compared to normoxic control offspring fed the control diet, and further, that weight gain was amplified in the hypoxic male offspring when fed a HFD from weaning. Indeed, the male offspring from hypoxic pregnancies fed a control diet had similar weight gain compared to the normoxic control males fed the HFD. As expected, control male offspring fed a HFD were heavier than the control male offspring fed a chow diet. Of note, we did observe the most robust difference in adult weights at 23 weeks of age although the differences were notable as early as 9 to 11 weeks of age.

Based on MRI, we observed an increase in total adiposity (peritoneal plus subcutaneous) in the hypoxic offspring fed the HFD compared to normoxic control offspring fed the control diet, with both the normoxic control offspring fed a HFD and the hypoxic offspring fed a control diet being intermediate, albeit not significantly greater compared to the normoxic offspring on a control chow diet (Fig 5). The total adiposity observed in the four groups corresponds with the observed differences in body weights by 23 weeks of age. We also found that while there were no differences between groups in subcutaneous fat volume, there was significantly greater peritoneal fat volume in both male offspring from hypoxic dams fed either the control diet or the HFD as well as the normoxic control offspring fed the HFD (Fig 5B). The hypoxic offspring on the HFD had the greatest peritoneal fat compared to the normoxic offspring on this diet, supporting the sustained increased food intake observed in the hypoxic offspring on the HFD.

The differences noted in the present study on post-weaning growth in male offspring exposed to gestational hypoxia compared to that of Reuda-Clausen et al [21] or Camm et al., [34] may reflect subtle differences in the degree of hypoxia, duration of hypoxic exposure or effects on maternal food intake that led to growth restricted fetuses. In the prior studies, a prolonged reduction in maternal food intake was noted that likely synergized with the hypoxic environment that led to the IUGR and the offspring growth phenotype. We did measure food intake in a separate study and found that our 12% oxygen hypoxia did reduce maternal food intake during the initial two days of hypoxia but by 17 days of gestation to term food intake was not significantly reduced. Based on this finding we performed a pair-feeding paradigm on normoxic dams from days 15 to term, reducing maternal nutrition to that observed in the hypoxic group during this period; the weights of male offspring from the pair-fed dams at 13 weeks of age were not different from normoxic control dams when fed a control chow diet. Thus, nutritional restriction may couple with hypoxia resulting in a different somatic growth and adipose deposition phenotype compared to hypoxia in the absence of in utero growth restriction.

### Effects of moderate gestational hypoxia on food intake post-weaning

We observed a significantly greater food intake from weeks four to seven post-birth in male offspring from hypoxic dams fed either the HFD or the control diet, as well as the male offspring from normoxic dams fed the HFD (Fig 3). From weeks eight through 23 (the final week of measuring food intake) only male offspring from hypoxic dams fed a HFD demonstrated an increased food intake compared to other groups. Our finding of an increased food intake in male offspring exposed to moderate gestational hypoxia differs from the finding reported by Rueda-Clausen et al [21] where Sprague-Dawley male offspring exposed to gestational hypoxia exhibited decreased energy intake when fed either a control diet (10% energy from fat) or a HFD (45% energy from fat). Again, the divergence in findings between the two studies may reflect the gestational hypoxic model in terms of severity of hypoxia or the combined presence of nutritional restriction leading to fetal IUGR. The study by Camm et al [34] did not record food intake. We did measure food intake in the male offspring from the pair-fed group during the first weeks post-weaning on a normal chow diet and noted no effect of pair-feeding on male offspring on daily food intake.

### Effects of moderate gestational hypoxia on motor activity and fear/anxiety behavior

To our knowledge, no other groups have examined the effect of gestational hypoxia on fear/anxiety in the offspring. The observations of Rueda-Clausen et al, [21] however, did find a reduced motor activity in the male offspring exposed to gestational hypoxia that may have contributed to the increased abdominal adiposity reported in that study as a result of decreased energy expenditure. We found no effects of gestational hypoxia on indices of anxiety-like behavior on the elevated plus maze nor indices of altered motor behavior (total entries, closed arm entries or rearing on maze) in the male offspring (Table 2) at adulthood (16 weeks of age). Although we realize that the elevated plus maze is not an optimal assessment of motor activity it is a widely used and accepted method of evaluating anxiety behavior that allows for a simultaneous evaluation of motor activity. Since we noted no changes in motor activity, further exploration of altered motor activity were not undertaken at the present time. Thus, it appears in our study that increased food intake and not decreased motor activity was a primary contributing factor to the noted increase in abdominal obesity in our offspring.

### Effects of moderate gestational hypoxia on adipose inflammatory markers (IL-1β, IL-6, and TNFα)

Increased inflammation in adipose, both from increased cytokine expression in adipocytes as well as increased recruitment of macrophages into the adipose, in particular in abdominal fat, is now recognized as a hallmark of diet induced obesity [42]. Of the major cytokines expressed in adipose tissue from obese subjects IL-1β, IL-6, and TNFα, have been linked with the development of insulin resistance. Although we did not address insulin resistance in the present study, we did examine both subcutaneous and peritoneal adipose expression (mRNA) for these cytokines. Curiously, in the control normoxic adult offspring fed the HFD only IL-6 was significantly elevated with trends for increased IL-1β and TNFα. This may indicate that a longer feeding period of the HFD was needed to induce a robust inflammatory state. In the adult male offspring from hypoxic dams, IL-6 was also increased with a similar trend for increased IL-1β and TNFα when fed the control diet and these offspring fed the HFD exhibited the greatest adipose inflammatory state with significant increases in all three cytokines measured. This reflects the degree of abdominal adiposity as measured by MRI. Thus, gestational hypoxia resulted in the highest indices of inflammation including increasing abdominal inflammation even when being fed a control, relatively low fat (10%) diet.

### Effects of moderate gestational hypoxia on the neonatal leptin surge, ARH (cFos), and αMSH projection fibers to the PVN in response to exogenous leptin at PN16

The increased appetite noted early post-weaning through at least week eight (for hypoxic and control offspring on the HFD and the hypoxic offspring on the control diet) and possibly several weeks longer likely contributes strongly to the noted increased adiposity in these groups (in particular peritoneal fat) suggesting dysregulation of hypothalamic neuroendocrine pathways regulating appetite and energy balance by gestational hypoxia. While hypoxia itself may play a direct role in disruption of this system, especially since the hypoxic challenge was during a window of hypothalamic nuclei differentiation and expansion, it is also possible that hypoxia affects this system indirectly via other systems. Previous studies in rodents have shown that during the first two weeks post-birth leptin plays a critical role in the establishment of hypothalamic neurocircuits governing appetite [24]. During this period in rodents, plasma leptin undergoes a so called ‘neonatal leptin surge’ [25, 26]. In the ob/ob mouse, which lacks leptin, hypothalamic neurocircuits governing appetite are severely underdeveloped and these animals become hyperphagic and obese as adults [24, 43, 44]. Similarly, administration of a leptin receptor antagonist during this period results in a similar hyperphagic and obese phenotype [29, 45]. Paradoxically, neonatal administration of leptin in rats leads to hypothalamic resistance to leptin, hyperphagia and increase adiposity later in life. Moreover, we previously reported in sheep that high altitude induced moderate gestational hypoxia resulted in elevated expression of perirenal adipose leptin and plasma leptin concentrations in the fetuses by near-term indicating that gestational hypoxia may impact developing adipose leading to excess leptin production [33].

Considering the critical role for leptin in development of hypothalamic neurocircuits governing appetite, we examined plasma leptin during the neonatal period in response to our gestational hypoxia paradigm. Male offspring from hypoxic dams exhibited a significant elevation in plasma leptin from days 6 through 14 post-birth. To address whether this increase in plasma leptin was impacting hypothalamic appetite neurons in the ARH, we examined both basal and exogenous leptin ARH neuronal activity at postnatal day 16 by immunostaining for c-fos. In the ARH of male offspring from hypoxic dams, the number of c-fos stained neurons in the ARH were significantly greater compared to normoxic offspring. In response to exogenous leptin however, only the normoxic offspring exhibited a significant increase in the number of c-fos labeled ARH neurons (compared to saline treated). The elevated leptin observed in the hypoxic offspring may result in the noted increased basal ARH activation observed. The decreased response to exogenous leptin in the hypoxic offspring may imply a limited capacity to respond to the acute increase in leptin from exogenous leptin delivery, or perhaps a decrease in the number of ARH neurons expressing leptin receptors. Alternatively, leptin transport across the blood brain barrier may be altered in the hypoxic offspring limiting leptin transport and thus ARH activation. In adult rodents with sustained elevated leptin associated with obesity, a profound desensitization to leptin is noted [46], thus a similar scenario may be occurring in these hypoxic offspring. We also observed a decrease in the density of αMSH fibers in the PVN. Thus, the altered ARH projection to other appetite centers (PVN) and/or continued insensitivity to plasma leptin may provide the mechanism for the noted increase in appetite in the hypoxic offspring leading to increased adiposity. Interestingly, leptin is a hypoxia inducible gene, however, the mechanism of gestational hypoxia leading to post-natal increases remains equivocal. Clearly, future studies will need to be performed to determine the exact role of the altered leptin surge per se we observed in response to moderate gestational hypoxia vs potential direct effects of gestational hypoxia on the programming of appetite dysregulation and obesity.

### Perspectives

Our model of moderate gestational hypoxia in the absence of IUGR allowed us to focus on effects on appetite, post-weaning growth on either a control or HFD and to identify mechanisms that could lead to perturbed appetite contributing to adult obesity. Our present findings showed 1) a significant increase in weight gain post-weaning in male offspring from hypoxic dams when compared to hypoxic male litter mates that were fed on the control diet, as well as male offspring from normoxic control pregnancies that were fed either the HFD or control diet, 2) our data demonstrated a significant increase in total adiposity (peritoneal plus subcutaneous) in the hypoxic offspring that were fed the HFD vs the normoxic control offspring on the control diet, and a significant increase in peritoneal fat volume in both male offspring from hypoxic dams fed either the control diet or the HFD as well as the normoxic control offspring fed the HFD, 3) in the adult male offspring from hypoxic dams, we also found that IL-6 was increased when fed the control diet, and when fed the HFD showed a significant increases in all three cytokines measured, 4) male offspring from hypoxic dams showed a significant increase in plasma leptin levels from days 6 through 14 post-birth, as well as showing a compromised developing rat pup ARH with decreasing αMSH projection fibers in the PVN. The changes we observed in the ARH and PVN postnatally in the rat pup that experienced gestational hypoxia has profound implications for other physiological systems in fetuses that are undergoing development and exposed to conditions of sustained moderate gestational hypoxia, and potentially programming in utero appetite dysregulation and future onset of adult obesity.
